# Impact of 8-Week Pilates Program on Lumbar Flexion–Relaxation Dynamics and Functional Outcomes in Women with Chronic Low Back Pain

**DOI:** 10.3390/jfmk11010085

**Published:** 2026-02-20

**Authors:** Ana Ferri-Caruana, Lluís Raimon Salazar-Bonet, Marco Romagnoli, Walter Staiano

**Affiliations:** 1 Department of Physical Education and Sport, Faculty of Science of Physical Activity and Sport, University of Valencia, C/ Gascó Oliag, 3, 46010 Valencia, Spain; 2 International University SEK, Quito 170151, Ecuador; 3 Department of Psychology, University of Southern Denmark, 5230 Odense, Denmark

**Keywords:** electromyography, asymmetry, core stability, trunk mobility, rehabilitation

## Abstract

**Objectives:** While Pilates exercise is commonly prescribed for chronic low back pain (CLBP), its effect on normalizing the lumbar flexion–relaxation ratio (FRR) remains unclear. This trial examined whether an 8-week Pilates exercise program (PEP) modifies FRR magnitude and side-to-side asymmetry in women with CLBP and explored associations with trunk kinematics, pain, and functional capacity. **Methods:** In a randomized controlled pre-test–post-test training design, ninety-six women with CLBP (55.8 ± 5.4 y) were allocated to a PEP group (n = 49) or a usual-care control group (n = 47). The PEP included two supervised 60-minute mat sessions per week over eight weeks. Surface electromyography of the right and left erector spinae and trunk flexion range of motion (TFRoM), measured via inertial sensors, were recorded during the standardized flexion–extension task pre- and post-intervention. Pain intensity (Visual Analog Scale) and functional capacity (Low Back Outcome Score, LBOS) were assessed concurrently. **Results:** Two-way repeated-measures ANOVA revealed no group × time interaction for global FRR (*p* = 0.454) or TFRoM (*p* = 0.745). FRR asymmetry increased by 11% in the PEP group (*p* = 0.033), with no change observed in the controls (*p* = 0.143). Compared to the controls, the PEP group exhibited a 30% reduction in pain (*p* = 0.003) and a 13.4% improvement in LBOS (*p* < 0.001) compared to the control group (all ps > 0.228). **Conclusions:** An 8-week Pilates intervention reduces pain and improves functional capacity in women with CLBP but does not restore lumbar extensor relaxation. The observed increase in FRR asymmetry may reflect compensatory or maladaptive redistribution.

## 1. Introduction

Clinical practice guidelines consistently recommend exercise therapy as a first-line treatment for chronic low back pain (CLBP) to improve function and reduce disability [1]. In this context, Pilates exercise has become widely adopted in both community fitness and rehabilitation settings for CLBP management, and is commonly recommended by health professionals [2]. Pilates-based programs emphasize controlled movement and core strengthening, making them a popular therapeutic option, especially among women with CLBP, who often participate in Pilates classes in high numbers [3].

Pilates is grounded in five foundational principles: centering, concentration, control, precision, flow, and breath; which collectively promote a mind–body approach to exercise. By encouraging postural awareness, diaphragmatic breathing, and precise motor control, these principles aim to optimize the activation and coordination of deep lumbo-pelvic musculature [4]. Notably, Pilates training has been shown to enhance core muscle engagement and strength in individuals with CLBP [5]. The focused breathing and muscle recruitment patterns in Pilates may thus confer neuromuscular benefits, potentially improving spinal stability and movement quality in this population [4,5].

A well-documented neuromuscular phenomenon related to trunk flexion is the flexion–relaxation *phenomenon* (FRP), characterized by a marked reduction or silence of the lumbar extensor muscles’ electromyographic (EMG) activity at end-range trunk flexion (6). In healthy individuals, the erector spinae (ES) muscles typically relax when the trunk is fully flexed, as posterior spinal ligaments and passive structures bear the load. Patients with CLBP, however, often exhibit an impaired or absent FRP; in other words, they maintain a higher than normal ES activity in full flexion [6]. To quantify this phenomenon, the flexion–relaxation ratio (FRR) is calculated as the ratio of extensor muscle EMG amplitude during the flexion movement to the EMG amplitude at full trunk flexion [7].

Clinically, a smaller FRR indicates a blunted or missing flexion–relaxation response. Indeed, meta-analytic evidence confirms that FRR values are significantly lower in individuals with chronic back pain compared to pain-free controls [6,8], reflecting an abnormal neuromuscular control and coordination of the trunk and hip in the presence of CLBP [8]. Encouragingly, flexion–relaxation behavior appears modifiable with therapy; prior studies have noted that the restoration of a normal FRP can occur following effective rehabilitation interventions [8].

There is, however, some controversy regarding how different exercise interventions influence the FRP/FRR in CLBP. Conventional physiotherapy or core stabilization programs have sometimes shown no measurable change in FRR despite improving symptoms [9,10]. For example, an 8-week lumbar stabilization training in people with CLBP significantly reduced pain and disability but did not alter the FRR, suggesting a persistence of the dysfunctional movement pattern. In contrast, more intensive or longer-duration exercise regimens have been associated with improvements in FRR metrics. Studies have reported increases in the FRR after targeted strengthening programs [11,12], as well as a higher proportion of patients regaining a normal flexion–relaxation response (and greater lumbar mobility) following comprehensive functional restoration rehabilitation [13]. These findings imply that, while basic or short-term exercise may be insufficient to reverse ingrained neuromuscular patterns, a more vigorous or prolonged training stimulus might restore the flexion–relaxation phenomenon in some CLBP patients. This mixed evidence warrants further investigation, particularly with exercise modalities like Pilates that emphasize motor control and could plausibly impact FRR.

Another important consideration in CLBP is the symmetry of muscle activation. An imbalance in activation between the right and left paraspinal muscles can lead to uneven spinal loading and has been implicated in persistent CLBP [14]. Accordingly, several studies have examined the flexion–relaxation *ratio asymmetry* (difference in FRR between the left and right ES muscles). Patients with nonspecific CLBP tend to exhibit greater side-to-side FRR asymmetry than asymptomatic individuals [14]. Rose-Dulcina et al. demonstrated a significant correlation (r ≈ 0.49) between FRR asymmetry of the ES and asymmetry in the lateral trunk range of motion, suggesting that uneven ES muscle relaxation is associated with restricted mobility in the corresponding direction [14]. Such findings reinforce the idea that lateralized neuromuscular deficits may contribute to the persistence of pain by chronically overloading one side of the spine. On the other hand, the relationship between the overall magnitude of the FRR and full trunk flexion range of motion (TFRoM) remains unclear, with some evidence indicating no straightforward association [14]. This gap in understanding highlights the need to explore how objective biomechanical measures like FRR and trunk flexibility interact in CLBP, and whether improvements in one translate to changes in the other.

Despite the popularity of Pilates in the management of chronic LBP, high-quality evidence of its efficacy and mechanisms is still emerging [15]. Recent systematic reviews and meta-analyses have concluded that Pilates exercise can yield significant reductions in pain intensity and disability in CLBP patients [3]. However, the current literature has largely focused on patient-reported outcomes, and there remains a limited understanding of Pilates’ effects on objective physiological or biomechanical markers of recovery [5]. No studies to date have examined changes in the flexion–relaxation ratio (or its asymmetry) because of Pilates training in individuals with CLBP. It also remains unknown whether any alteration in FRR corresponds with improvements in clinical outcomes such as pain or functional capacity in this context.

In light of the above considerations, the present study was designed to evaluate the effect of an 8-week Pilates exercise program on the lumbar flexion–relaxation ratio and its side-to-side asymmetry in women with chronic low back pain. We hypothesized that the Pilates intervention would increase the FRR (indicating an enhanced flexion–relaxation response) and concurrently decrease the FRR asymmetry of the erector spinae. A secondary aim was to explore the relationships between FRR outcomes and full TFRoM, pain intensity, and self-reported functional capacity in a female population.

## 2. Materials and Methods

### 2.1. Participants and Design

This randomized controlled trial (ID: NCT05264311) allocated participants into two groups: an experimental group (EG) receiving a Pilates exercise program (PEP) and a control group (CG) serving as a comparison.

**Figure 1:**
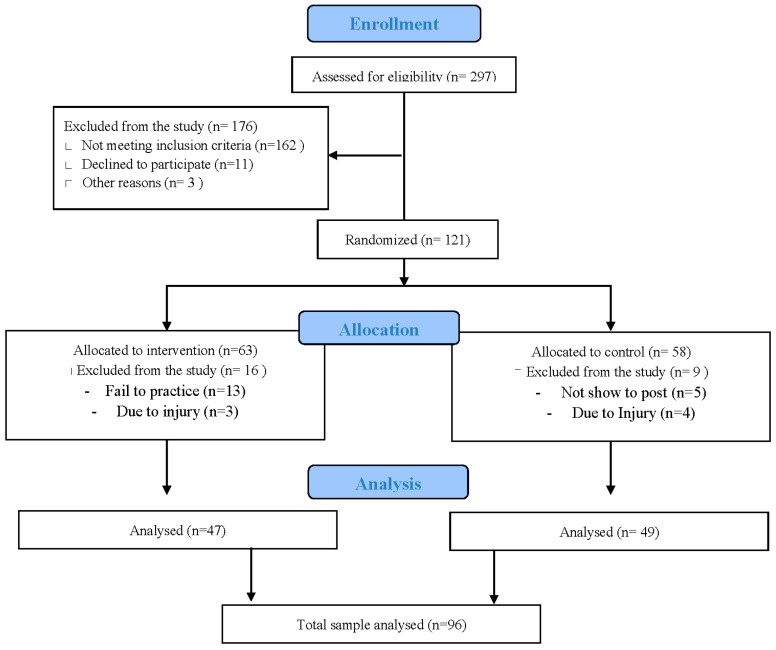
Flow Diagram for subject eligibility, randomization, dropout rate and number of subjects of final analysis.

A total of 121 female participants (age 55.8 ± 5.4 years) were recruited through flyers and posters placed at local sports centers, physiotherapy clinics, and general practitioner offices in Valencia. Interested individuals contacted the research coordinator, completed a telephone screening, and then attended an in-person evaluation where eligibility was confirmed through a clinical interview and ODI assessment (Figure 1 Flow Diagram). Participants meeting the inclusion criteria were randomly assigned to either the experimental or control group. Of the 121 initially enrolled, 25 were lost to follow-up: 14 from the intervention and 11 from the control group. The primary reasons included unrelated illness (n = 8), relocation (n = 6), lack of availability (n = 9), and one voluntary withdrawal due to perceived discomfort (n = 1). No adverse events or exercise-related injuries were reported throughout the trial. Of the final sample (n = 96), 25 of the dropout participants (26.0%) reported radicular symptoms at baseline, with no significant difference in their distribution between the experimental (13 of 49; 26.5%) and control groups (12 of 47; 25.5%) (*p* = 0.91) (Figure 1 Flow Diagram). Subgroup analyses showed no interaction effect between symptom type and outcomes.

Participants were randomly assigned to either group using a computer-generated randomization sequence (block size of 4), managed by an independent researcher not involved in recruitment or outcome assessment. Allocation was concealed using sequentially numbered, opaque, sealed envelopes. Outcome assessors and data analysts were blinded; due to the nature of the intervention, instructors and participants were not blinded. Chronic low back pain (CLBP) was defined as pain localized below the costal margin and above the inferior gluteal folds, with or without leg pain, persisting for more than 12 weeks (1). Inclusion criteria comprised lumbar or lumbosacral pain, with or without radicular symptoms, persisting for at least six months; an Oswestry Disability Index (ODI) score > 6/50 [16]; and no back treatment in the preceding three months. Exclusion criteria included body mass index (BMI) > 30 kg/m^2^; prior surgery involving the pelvis, spine, or lower extremities; scoliosis; systemic or degenerative diseases; neurological deficits unrelated to back pain; pregnancy; and hypertension [17].

Ethical approval was granted by the University of Valencia (Spain) and the International University SEK Quito (Ecuador) according to the declaration of Helsinki, and all participants provided informed written consent.

### 2.2. Procedures

All assessments were conducted pre- (T0) and post- (T9) the 8-week intervention in the physiotherapy laboratory where the PEP was administered. Environmental conditions (temperature, humidity) were monitored; 24 h prior to each session, participants adhered to standardized routines for sleep, diet, hydration, supplementation, medication and physical exercise.

Participants performed a standardized flexion–extension task: standing with feet shoulder-width apart and arms at their sides, they bent forward maximally over 4 s, relaxed fully flexed for 4 s, extended to upright standing over 4 s, and stood quietly for 4 s. This sequence was repeated three times, paced by a metronome [18]. Participants were instructed to keep knees extended, avoid abdominal contraction, and maintain head flexion to minimize cervical movement. Full trunk flexion range of motion (TFRoM) was recorded via an EMG inertial sensor affixed at the T3 vertebra with kinesio tape, calculating directly the average angular displacement across three trials [19].

The PEP consisted of 1-hour sessions twice weekly for 8 weeks, led by two Pilates professionals averaging 6.5 years’ experience. The program, previously published [20], emphasized core stability, posture, breathing, flexibility, strength, and muscle control, with a particular focus on pelvic–lumbar region stabilization through conscious trunk muscle activation. Exercises included the hundred, rolled up, single leg circles (bent leg), spine stretch, rolling like a ball, and single leg stretch. Each exercise involved 4 repetitions of 30 s, separated by 2-minute rest intervals. To complete 60 min, “superman” and double leg bridge exercises were added.

Prior to commencing the PEP, participants received a 1-hour introductory session to train core muscle activation, targeting isometric contraction of the transversus abdominis, pelvic floor, and multifidus during diaphragmatic exhalation. The protocol was supervised and individually adjusted based on participants’ ability to recruit target muscles and reduce postural compensations. Pain intensity was monitored before, during, and after sessions. All exercises were performed on ≥ ¾-inch-thick rubber mats. Attendance was recorded weekly.

The control group continued with usual care, defined as maintaining habitual physical activity and healthy routines. No structured exercise program or new physiotherapy was permitted during the 8-week period. The control group was contacted biweekly to maintain engagement and confirm absence of protocol deviation. No cointerventions were permitted, and both groups maintained usual lifestyles; nonsteroidal anti-inflammatory drug (NSAID) use was allowed and recorded. Biweekly telephone calls and training diaries ensured no unusual physical activity outside the protocol.

### 2.3. Measures

Functional capacity was assessed using the Spanish-adapted Low Back Outcome Score (LBOS) questionnaire [21]. Pain intensity was evaluated via a 10 cm Visual Analog Scale (VAS), anchored at “no pain” (0 cm) and “worst pain” (10 cm). Both LBOS and VAS were measured at T0 and T9.

Surface electromyographic activity was recorded bilaterally from the erector spinae (ES) using a portable 2-channel Shimmer device (Realtime Technologies Ltd., Dublin, Ireland) with 16-bit analog-to-digital conversion at 1024 Hz sampling frequency. EMG signals were monitored via the mDurance software (MDurance Solutions S.L., Granada, Spain) on Android and stored in a cloud server. Signals underwent automatic fourth-order Butterworth band-pass filtering between 20 and 450 Hz, employing a 20 Hz high-pass cutoff to minimize movement artifacts while preserving signal integrity [22]. Root mean square (RMS) values were computed from filtered signals, averaged over the duration of each movement phase. Skin was prepared by shaving, abrading, and cleaning with alcohol. Bipolar pre-gelled Ag/AgCl surface electrodes (MedCaT B.V., Doorndistel, Spain) were placed following SENIAM guidelines [22]. ES muscle asymmetry was calculated as the mean RMS difference between right and left sides across the three flexion–extension tasks. The FRR was computed by dividing the maximal EMG amplitude during flexion by the minimum amplitude at full flexion [12]. Means from the three trials were used for each muscle and participant. A higher FRR indicates greater muscle relaxation. For each participant, a global FRR was calculated by averaging the left and right side FRR values. FRR asymmetry was defined as the absolute difference between right and left ES FRR values.

### 2.4. Statistical Analysis

Data are presented as mean ± standard deviation (SD) unless otherwise noted. Assumptions of normality and homogeneity of variance were verified using Shapiro–Wilk and Levene tests, respectively. A priori power analysis using MorePower 6.0.4 indicated that a sample size of 96 would provide 80% power to detect a medium effect size (f = 0.27, η^2^p = 0.05) for group × time interactions in pain (VAS), functional capacity (LBOS), and FRR metrics, assuming α = 0.05. These primary outcomes were selected based on prior literature emphasizing their clinical and mechanistic relevance in CLBP rehabilitation trials.

Baseline group differences were tested using independent *t*-tests. Two-way repeated measures ANOVA (GROUP × TIME) evaluated intervention effects across dependent variables. Significant main or interaction effects were further explored using Bonferroni post hoc tests. Effect sizes were calculated as partial eta squared (η^2^p), interpreted as small (0.01), medium (0.06), or large (0.14). Statistical significance was set at *p* ≤ 0.05. Statistical analyses were conducted using SPSS v27 (SPSS Inc., Chicago, IL, USA).

## 3. Results

### 3.1. Demographics and Baseline

The age, height, weight and BMI were not significantly different between groups at baseline (Table 1). There were no differences at baseline in the variables of the FRR (*p* = 0.708), FRR asymmetry (*p* = 0.973), full TFRoM (*p* = 0.450), pain intensity (*p* = 0.522) and functional capacity (*p* = 0.611). There were no significant differences between groups at baseline in the low back pain (LBP) disability index ODI (*p* = 0.223); the mean scores for CG and EG were 15.4% and 14.2%, respectively, higher than the minimal score allowed to participate in the study (14%, equivalent to 7/50). None of the participants missed more than one session, as required to be included in the analysis. All participants from each group were included in all analyses. The dropout rate was 20.7%, with no significant between-group difference in attrition (*p* = 0.61). No participant reported adverse reactions or serious events related to the intervention.

### 3.2. Effect of the Pilates Exercise Program on FRR and FRR Asymmetry

**Figure 2:**
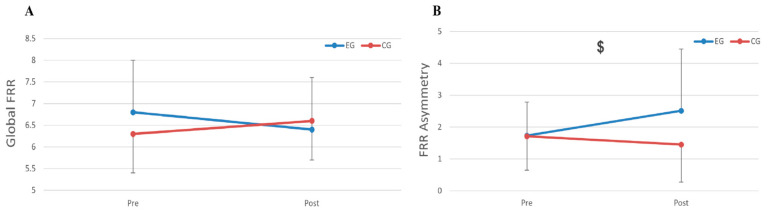
(**A**) Changes in global flexion–relaxation ratio (FRR) pre- to post-intervention in the experimental group (EG) and control group (CG). No significant group × time interaction was observed, indicating that the Pilates intervention did not alter overall FRR magnitude. (**B**) Changes in flexion–relaxation ratio (FRR) asymmetry pre- to post-intervention in the experimental group (EG) and control group (CG). A significant group × time interaction ($, *p* < 0.05) was observed for FRR asymmetry, with the EG showing increased asymmetry post-intervention compared to CG, suggesting potential imbalances in neuromuscular control despite unchanged global FRR values.

There was no significant GROUP × TIME interaction (*p* = 0.454, η^2^p = 0.020) or main effects (all ps > 0.356, η^2^p < 0.030) for FRR (Figure 2A). However, there was a significant GROUP × TIME interaction for FRR asymmetry (*p* = 0.034, η^2^p = 0.137) (Figure 2B). Follow-up tests revealed that the EG showed a significant (*p* = 0.033) increase in FRR asymmetry at post-test compared to baseline, while no significant differences were found in the CG from baseline to post-test (*p* = 0.143). In the EG, the mean left-side FRR increased from 1.6 ± 0.4 at baseline to 1.7 ± 0.4 post-intervention, while the right-side FRR rose from 1.7 ± 0.5 to 1.9 ± 0.5. In the control group, the left-side FRR was 1.6 ± 0.3 at baseline and 1.5 ± 0.3 post-intervention, and the right-side FRR was 1.6 ± 0.4 and 1.6 ± 0.4, respectively. This divergence between sides in the EG explains the greater FRR asymmetry observed post-intervention.

### 3.3. Effect of the Pilates Exercise Program on Full Trunk Flexion Range of Motion (TFRoM), Pain Intensity and Functional Capacity, and Their Relationship with FRR

**Figure 3:**
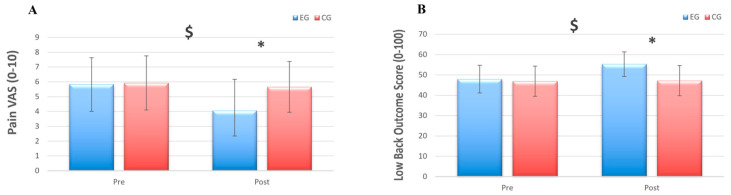
(**A**) Pain intensity assessed via Visual Analog Scale (VAS). A significant group × time interaction ($) and post hoc difference (*) indicated a clinically meaningful reduction in pain following the intervention in the experimental group (EG) only compared to the control group (CG). (**B**) Low Back Outcome Score (LBOS) improved significantly in the experimental group (EG) post-intervention ($, *p* < 0.05), while no change was observed in the control group (CG). These findings suggest functional improvements alongside pain reduction in the intervention group.

Full TFRoM did not show any significant interaction (*p* = 0.745, η^2^p = 0.004) or main effects (all ps > 0.193, η^2^p < 0.020). There was a significant GROUP × TIME interaction for pain intensity (*p* = 0.003, η^2^p = 0.268) (Figure 3A). Follow-up tests revealed that participants in the EG rated the pain intensity significantly lower (*p* = 0.021) (30%) at post-test compared to baseline, while participants in the CG did not show any significant difference (*p* = 0.372) from baseline to post-intervention. Pain scores (VAS) in the Pilates group decreased from 5.9 ± 1.8 at baseline to 4.1 ± 1.3 post-intervention, whereas control group scores changed from 5.7 ± 1.8 to 5.5 ± 1.6. There was a significant GROUP × TIME interaction for functional capacity (*p* ˂ 0.001, η^2^p = 0.570) (Figure 3B). Follow-up tests revealed that the EG significantly (*p* ˂ 0.001) increased functional capacity (13.4%) at post-test after the PEP intervention, while no significant difference was reported for functional capacity in the CG (*p* = 0.228).

## 4. Discussion

The present controlled trial investigated whether an 8-week Pilates exercise program (PEP) could normalize the flexion–relaxation ratio (FRR) and its side-to-side asymmetry in the erector spinae (ES) during maximal trunk flexion–extension in women with chronic low back pain (CLBP). The secondary aims were to determine the effects of PEP on full trunk flexion range of motion (TFRoM), pain intensity and functional capacity, and to explore how these clinical outcomes relate to FRR behavior. In brief, the intervention reduced pain and improved self-reported function yet produced no change in the global FRR, worsened FRR asymmetry, and left the TFRoM unaltered. These findings refine the current understanding of the neuromuscular mechanisms underpinning Pilates efficacy in CLBP and highlight key moderators—dose, motor-learning stage, psychological load and age—that may dictate whether FRR is a modifiable target. The training had strengths that included concealed allocation, blinded data analysis, high adherence (> 95%), and comprehensive EMG profiling.

Pilates is reputed to enhance deep-core recruitment and optimize lumbo-pelvic motor control through principles of centering, precision and breath regulation. Meta-analytic syntheses show that it achieves superior pain and disability reductions versus general strengthening or stretching in CLBP [23]. At the electromyographic level, two recent systematic reviews confirm modest gains in core-muscle activation efficiency following ≥ 12 weeks of Pilates [5]. Yet our 8-week PEP did not alter FRR—an objective proxy for lumbar extensor relaxation. The null effect replicates Shahvarpour et al.’s stabilization trial in which eight weeks was likewise insufficient to shift FRR despite symptomatic relief [10].

Several mechanistic explanations emerge. First, motor-learning data indicate that novices need at least ten to twelve weeks of practice before complex neuromuscular patterns, such as deep-core pre-activation and timely extensor silencing, are automatized [6]. Second, age modulates FRR plasticity; a 2024 cross-sectional analysis showed that FRR restoration slows markedly after 40 years, with older CLBP patients displaying entrenched co-contraction strategies that resist short-dose training [6]. Our mid-fifties cohort thus faced a double barrier of limited dosage and age-related motor inflexibility. Third, FRR gains are stronger when exercises are coupled with real-time surface EMG (sEMG) biofeedback, as demonstrated by Neblett et al. [11] and by recent RCTs employing visual biofeedback during core drills, which report ≥ 30% FRR increases within six weeks [24]. The absence of biofeedback in our protocol may therefore have hampered neural recalibration.

Marshall et al. [12] documented FRR increases after trunk-conditioning but lacked a control arm and baseline verification of FRR impairment; improvements might therefore reflect a regression to the mean. More compelling are dose–response trials in which 12- to 16-week Pilates or combined core-strengthening programs produced a significant FRR enhancement and spinal unloading [6,25]. Compared with these designs, our shorter regimen may have delivered an insufficient cumulative training stimulus (<16 h total). The emerging consensus from dose-finding experiments is that ≥ 24 h of targeted core work is the minimum to reverse chronic co-activation patterns [25].

A striking observation was the 11% rise in FRR asymmetry post-intervention. ES side-to-side imbalance is clinically meaningful—large asymmetry predicts greater spinal shear forces, facet joint loading and recurrence risk [14,26,27]. Very few interventional studies have tracked this metric; Rutkowska et al. recorded non-significant drift after four weeks of core training [28]. Our negative asymmetry shift may signal that participants subconsciously protected the more painful side by preferentially stiffening it, thereby transferring the workload to the contralateral ES. Such asymmetric guarding aligns with evidence linking pain-related fear and protective trunk stiffness with altered FRR patterns [29]. A 2024 study further demonstrated that FRR asymmetry is tightly coupled to multi-segmental kinematic irregularities in CLBP [25], supporting the biomechanical plausibility of our finding.

Because our asymmetry index is based on the *difference* between left and right FRR, it can increase when one side changes more than the other (inter-limb divergence), even if the bilateral mean FRR remains relatively unchanged.

Clinically, this suggests that symmetrical bilateral drills, a hallmark of Pilates mat routines, may allow covert protective biases to persist unless instructors explicitly cue side-specific relaxation or integrate unilateral tasks. Immersive virtual-reality paradigms manipulating visual trunk-flexion feedback successfully reduced FRR asymmetry and pain within four sessions [14], indicating that augmenting Pilates with visual biofeedback could correct side-dominant compensation.

Although FRR asymmetry increased following the intervention, the absence of pain lateralization data prevents mechanistic inferences about a protective strategy on the more painful side. The observed pattern may reflect heterogeneous or task-dependent neuromuscular adaptations, potentially representing a compensatory or maladaptive redistribution of trunk extensor activation rather than a confirmed protective mechanism. Accordingly, these findings should be interpreted as hypothesis-generating and warrant replication with an explicit assessment of pain lateralization and movement asymmetries.

The lack of TFRoM change replicates Shahvarpour et al.’s observation in which pain relief occurred alongside static mobility [10]. It contrasts with trials showing Pilates-driven TFRoM gains when baseline functional limitation is moderate to severe (e.g., ODI > 20%) [2,17,30,31,32]. Our participants reported low baseline disability (ODI ≈ 11/50), offering limited headroom. Moreover, recent biomechanics work dissociates FRR improvement from TFRoM increments, underscoring that lumbar extensor relaxation can recover without reaching larger flexion angles, provided biofeedback is used [25]. Thus, the static TFRoM in our sample does not necessarily indicate a failure of motor control adaptation; rather, it may reflect ceiling effects or persistent fear-driven movement restriction.

Pain intensity dropped by 30% and the LBOS improved 9.8%, mirroring pooled Pilates effects of 16–54% pain reduction in meta-analyses (2, 53) and in recent trials targeting older adults [33] and sub-acute low back pain (LBP) [2]. The weak correlation between FRR and functional change echoes Watson et al.’s conclusion that FRR restoration predicts disability only when neuromuscular retraining is sufficiently intensive [34]. Our data therefore align with a dual-mechanism model: short-dose Pilates provides analgesia via central pain-modulating mechanisms (e.g., enhanced cortical inhibition, mindfulness), yet deeper neuromuscular reorganization of the FRR requires a higher volume, biofeedback and psychological readiness.

Fear-avoidance beliefs predict a reduced lumbar flexion and heightened ES co-contraction in both pain-free and CLBP cohorts [29,35]. They diminish following Pain–Neuroscience Education combined with Pilates and partially mediate disability gains [33]. We did not measure fear avoidance, yet the rise in FRR asymmetry alongside pain reduction suggests persistent protective stiffness. Future trials should incorporate validated fear-avoidance and self-efficacy scales to parse psychological from biomechanical drivers of FRR change and to test whether targeting maladaptive beliefs accelerates neuromuscular normalization.

### Limitations and Future Directions

We acknowledge some limitations of this study. The limitations mirror those acknowledged earlier: female-only sampling limits generalizability; single-sensor TFRoM failed to isolate the pelvic contribution; and more in-depth psychological variables were not captured. Additionally, a lack of long-term follow-up precludes conclusions about the durability of pain relief or asymmetry progression. An additional methodological limitation impacting on the interpretation of our results is the lack of specific information regarding pain lateralization in the subjects included in the study. While the significant increase in FRR asymmetry in the Pilates group is interpreted as a possible protective motor adaptation on the more painful side, we did not collect data correlating the change in FRR asymmetry with the symptomatic side of the pain. We acknowledge that this omission limits our ability to conclusively interpret whether the increased asymmetry is the result of a lateralized protection bias driven by pain. Therefore, future trials must explicitly measure pain lateralization to correlate it with post-intervention changes in FRR asymmetry and thus validate the hypothesis of this protective mechanism. The usual care condition allowed NSAID use. Given the evidence that NSAIDs can provide modest pain relief in low back pain, differential or unreported use may have influenced pain outcomes and reduced detectable group differences [36]. Similarly, physical activity was not quantified; variations in habitual activity or exercise outside the intervention may influence pain/disability and analgesic use, representing an additional source of uncontrolled variability [37].

To delineate the causal chain from Pilates to neuromuscular normalization, future trials should employ: multi-segment motion capture to link FRR asymmetry with spine–pelvis kinematics [25]; wearable sEMG-triggered feedback delivered during home practice to increase dose and ecological validity; and stratification by age, chronicity, and fear-avoidance to tailor dosage and cognitive adjuncts.

A longer follow-up could be an option to test whether early asymmetry correction predicts recurrence. Moreover, it could employ health–economic modeling to weigh the cost of extended or feedback-augmented programs against the societal burden of CLBP. Further research is warranted to optimize the dose, integrate behavioral components [38,39] and verify the long-term biomechanical benefits of Pilates-based rehabilitation.

## 5. Conclusions

An 8-week Pilates program produced clinically meaningful gains but did not improve global FRR and increased FRR asymmetry in middle-aged women with CLBP. These data suggest that, while Pilates confers short-term symptom relief, neuromuscular normalization requires a higher training volume, targeted biofeedback and psychological engagement. The observed increase in FRR asymmetry should be interpreted cautiously; without pain lateralization data, its clinical meaning remains uncertain and may reflect compensatory or maladaptive neuromuscular redistribution. Health professionals should monitor side-dominant protective patterns during bilateral Pilates drills and integrate strategies that explicitly retrain symmetrical extensor relaxation.

## Figures and Tables

**Table 1:** Subjects’ anthropometric measurements, functional capacity and pain status.

	CG (n = 47)	EG (n = 49)	
	M ± SD	M ± SD	*p*
Age (years)	56.1 ± 5.4	55.5 ± 4.6	0.62
Height (cm)	158.4 ± 5.6	162.5 ± 5.9	0.81
Weight (kg)	65.8 ± 5.0	63.2 ± 4.6	0.17
BMI (kg/m2)	25.5 ± 2.2	24.4 ± 2.0	0.23
ODI (/50)	14.1 ± 4.3	12.1 ± 4.5	0.22
VAS (/10)	5.7 ± 1.8	5.9 ± 1.8	0.76
LBOS (/75)	47.8 ± 7.4	48.1 ± 6.8	0.52
LBP prior the study (weeks)	19.2 ± 6.3	18.9 ± 7.5	0.44

EG = experimental group; CG = control group; n = number of subjects; M = mean; SD = standard deviation; BMI = body mass index; cm = centimeter; kg = kilogram; ODI = Oswestry Disability Index; VAS = Visual Analog Scale; LBOS = Low Back Outcome Score; LBP = low back pain.

## Data Availability

Due to EU General Data Protection Regulation original data cannot be shared.
